# Plasmonic Sensing Assay for Long-Term Monitoring (PSALM) of Neurotransmitters in Urine

**DOI:** 10.1021/acsnanoscienceau.2c00048

**Published:** 2022-12-24

**Authors:** Wei-Hsin Chen, Wenting Wang, Qianqi Lin, David-Benjamin Grys, Marika Niihori, Junyang Huang, Shu Hu, Bart de Nijs, Oren A. Scherman, Jeremy J. Baumberg

**Affiliations:** † NanoPhotonics Centre, Cavendish Laboratory, University of Cambridge, J J Thomson Avenue, Cambridge CB3 0HE, U.K.; ‡ Melville Laboratory for Polymer Synthesis, Department of Chemistry, University of Cambridge, Lensfield Road, Cambridge CB2 1EW, U.K.

**Keywords:** SERS, sensing, neurotransmitters, dopamine, urine, self-assembly

## Abstract

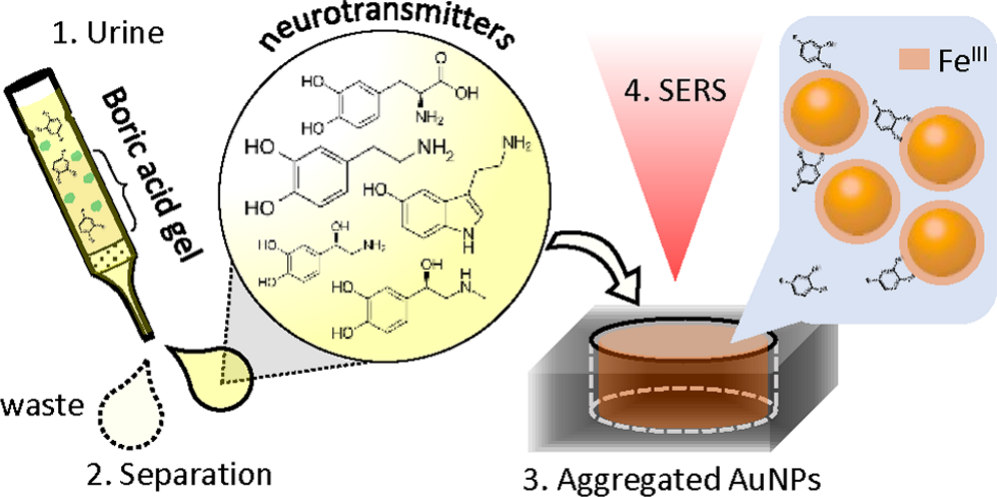

A liquid-based surface-enhanced Raman spectroscopy assay termed PSALM is developed for the selective sensing of neurotransmitters (NTs) with a limit of detection below the physiological range of NT concentrations in urine. This assay is formed by quick and simple nanoparticle (NP) “mix-and-measure” protocols, in which Fe^III^ bridges NTs and gold NPs inside the sensing hotspots. Detection limits of NTs from *PreNP* PSALM are significantly lower than those of *PostNP* PSALM, when urine is pretreated by affinity separation. Optimized PSALM enables the long-term monitoring of NT variation in urine in conventional settings for the first time, allowing the development of NTs as predictive or correlative biomarkers for clinical diagnosis.

## Introduction

The measurement of chemical messengers as a means to assess the function of organs or tissues has become the basis for diagnostic or functional indicators in clinical practice.^1^ Despite a historical absence of relevant biomarkers in the realm of clinical psychiatry, the significant contribution of neurotransmitters (NTs) to not only neurological functioning but also endocrinological and immunological action has expanded the use of NTs as a primary target for the development of predictive or correlative biomarkers of nervous system function.

Dopamine is an NT of great clinical importance for motor function and motivational behavior. Its dysfunction is involved in many psychiatric disorders, including drug addiction,^2^ schizophrenia,^3^ and psychiatric conditions.^4^ Norepinephrine and epinephrine are involved in the autonomic nervous system. In the hypothalamus, amygdala, and locus coerulus, increased norepinephrine release has been associated with anxiety, while epinephrine plays a role in the fight-or-flight response by increasing the heart rate, vasodilatation, pupil dilation, and blood sugar.^5^ However, their concentrations in body fluids are only 0.01–1 μM^6^ while coexisting with many types of interfering molecules such as amino acids, nucleic acids, glucose, urea, cysteamine, etc. It is thus important to measure NTs accurately while at the same time distinguishing NTs from other molecules in the biofluids.

Previous approaches to NT sensing and monitoring can be classified into several categories:^1^ (1) nuclear medicine tomographic imaging, including positron emission tomography^7^ and single-photon emission computed tomography.^8^ These have a high spatial resolution but require expensive infrastructure and complex manipulation, giving limits of detection (LODs) for dopamine of 200 nM.^9^ (2) Electrochemical detection, including voltammetry^10^ and amperometry.^11^ These are notionally easy to implement in implantable devices in addition to being cost-effective, with an LOD for dopamine of ∼50 nM.^12^ However, due to the low selectivity and the complexity of in vivo chemical monitoring, their usage for real-world applications requires further advances. (3) Analytical chemistry techniques, including high-performance liquid chromatography and mass spectroscopy, are very sensitive, with LODs for L-glutamate, GABA, dopamine, serotonin, and 5-hydroxyindole acetic acid of 0.4–1.3 nM.^13^ However, they require expensive equipment and complex manipulation and are time consuming.^1^ (4) Microdialysis, which is an invasive detection technique, but must be used alongside other techniques^14,15^ to monitor amines, amino acids, NTs, neuropeptides, and acetylcholine in the human brain.^16,17^ (5) Optical sensing, including fluorescence, chemiluminescence, optical fiber-based biosensors, and colorimetry.^14^ These are suitable for miniaturization thanks to rapidly advancing optoelectronic and microfabrication technologies, emerging as promising for NT detection with high accuracy. However, their use is challenging due to the sparsity of NTs and their admixture with other molecules.

These approaches are so far limited by their sensitivity, selectivity, and complexity. It is thus critical to explore novel sensing approaches, which combine high selectivity, sensitivity, and low manipulation complexity for NTs. Vibrational spectroscopies (such as Raman) can identify molecules based on their characteristic resonant peaks and are useful for detecting different NTs even though some of their structures are similar. Surface-enhanced Raman spectroscopy (SERS) uses optical interactions with metals to enhance the Raman scattering by molecules adsorbed in the proximity of optical “hotspots” on a nanostructured metal surface.^18,19^ For instance, aggregated gold nanoparticles (AuNPs) can show enhancement factors reaching one billion,^20^ greatly enhancing LODs. Previous SERS studies of NTs include a spread spectrum technique to enhance the signal-to-noise ratio,^21^ looking at NTs in cells,^22^ competitive binding of DA and albumin in cerebrospinal fluid,^23^ specific aptamer binding of DA in a Au and graphene construct,^24^ and NT SERS from different metals.^25^

NTs can be selectively bound to these hotspots through different surface functionalizations. In particular, multivalent iron ions (*e.g.* Fe^III^) can form bidentate complexes with a catechol through the Fe–O coordination bond.^26,27^ Catecholamine NTs (containing this catechol structure) therefore adsorb onto Fe^III^-modified metal surfaces, bringing them into the SERS hotspots to give enhanced Raman signals. For instance, silver nanoparticles have been modified with Fe nitrilotriacetic acid to bind dopamine for SERS sensing achieving LODs of <1 nM.^28^ Citric acid was also used with an Fe^III^ complex to bind dopamine onto AuNPs for low-concentration SERS detection.^29^ Citric acid molecules can also simultaneously bind to Fe^III^, which is important to note since these are typically surface-bound on AuNPs in suspension for charge stabilization. Through titration, Franz *et al*.^30^ found that dopamine binds to Fe^III^ as mono-, bis-, and tris-complexes, depending on the pH. Systematic studies show that the predominant ferric citrate species at neutral pH is the mono iron di-citrate [Fe(Cit)_2_]^3–^ for iron/citrate molar ratios below 1:10, while above this, oligomeric species become appreciable.^31^ Overall, this work suggests that it is highly promising to bind NTs to citrate-capped Au surfaces modified with Fe^III^ by forming a complex of NTs–Fe^III^–citrate–Au.

In this paper, we demonstrate a simple and efficient “mix-and-measure” method to form a liquid sensing platform termed a plasmonic sensing assay for long-term monitoring (PSALM), which shows great potential for full automation. PSALM can perform fast detection of NTs at physiological concentrations in water (as a control) or in urine. With the approach presented in this paper, PSALM could potentially open up opportunities for home-based health monitoring, for instance in quantitatively tracking NTs to provide useful information for psychiatric professionals in diagnosing drug prescriptions for mental illness or stress or in drug regime compliance. Combining analyte, AuNPs, NaCl (to induce aggregation), and an Fe^III^ salt (to enhance the signal strength through coordination complexation) in different sequences is shown to give limits of detection down to ∼1 nM, 100-fold less than physiological concentrations. We discuss the optimization of this assay and the role of Fe^III^ in sequestering the analyte NTs in the plasmonic hotspots of the AuNP aggregates, enhancing their fingerprint SERS signals. We find that it is crucial to avoid interference between Fe^III^ and functional groups of unwanted elements in urine. To achieve this, the pretreatment of urine using affinity separation is adopted to elute out the targeted NTs for subsequent SERS measurements.

We compare two distinct protocols (“*PreNP*” and “*PostNP*”), which differ in the order of steps for forming PSALM, affecting both the LOD and the NT SERS intensities. Characterizing the complexation of Fe^III^ and dopamine (DA) in different pH values suggests that Fe^III^DA_2_ dominates in *PostNP* PSALM detection, while in *PreNP* PSALM it is the monomeric Fe^III^DA that is seen. Protonation of the NT’s hydroxyl bonds occurs when the solution is in an acidic condition, which is competitive to hydroxyl’s binding to Fe^III^, leading to a decrease in SERS. Full optimization is thus crucial for PSALM sensing, which improves the ability of NTs to bind to the surface of AuNPs and migrate to the vicinity of the hotspots.

This assay is formed from suspended AuNP aggregates in water, in which the optical field is enhanced in the resulting nanoscale metallic gaps (spaced <1 nm apart by the citrate ligands on the AuNPs).^27^ This greatly enhances the SERS signals of molecules in the proximity of the gaps. A great many prior works have explored different ways to make such SERS substrates;^32^ however, for the applications discussed here, which require repeated production of identical sensors with no contamination from preproduction/storage protocols and to be cheap enough for frequent testing, a solution-based colloidal assembly is preferred. Because of the strong SERS enhancements, signals in solution are strong enough without the need to dry down and concentrate the colloids/analytes, as frequently reported in the literature.^32^ This means time-consuming measurements, typically required to average over substrate nonuniformity, are not required as the colloidal stability of these aggregates means that during the measurement time many aggregates diffuse through the laser spot, providing the required signal averaging.^28^ We also explored different aggregation techniques and protocols and opt for salt aggregation as this prevents confounding factors in the molecular complexation mechanisms and does not introduce additional Raman peaks in the NT detection (Figures S1 and S2).

## Results and Discussion

### Role of Fe^III^ in Different Protocols of PSALM for Sensing Dopamine

**Figure 1:**
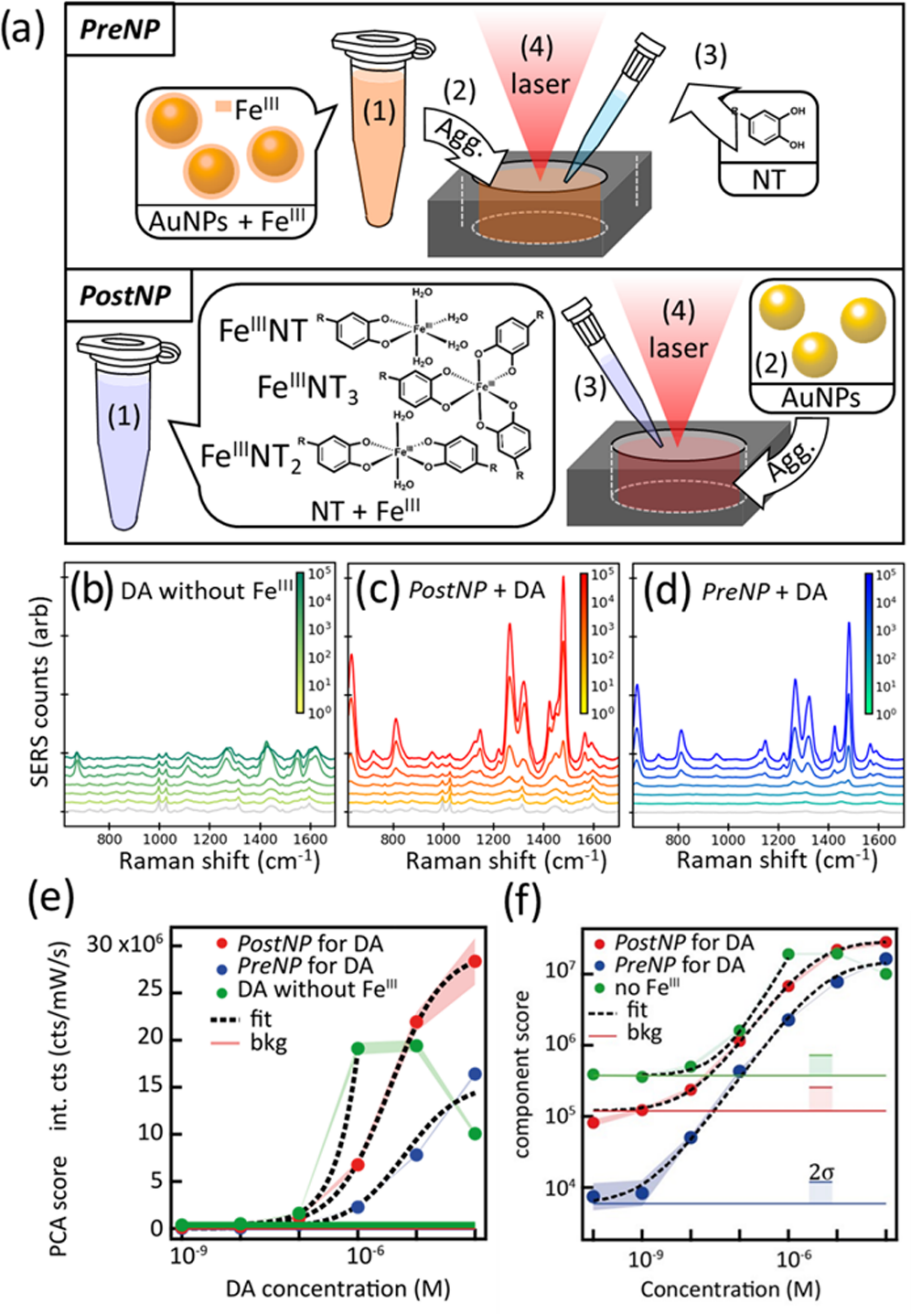
(a) Two PSALM protocols used for sensing NTs: *PreNP*: (1) AuNPs preincubated with Fe^III^, (2) then aggregated using NaCl, (3) NT samples added and mixed thoroughly. *PostNP*: (1) NTs preincubated with Fe^III^, creating Fe^III^NT, Fe^III^NT_2_, and Fe^III^NT_3_ complexes, (2) AuNPs separately aggregated using NaCl, and then (3) Fe^III^NT_*n*_ complexes added to the aggregate solution and mixed thoroughly. In both cases, the final step (4) focuses a 785 nm laser into the solution to obtain SERS. (b–d) Collected baseline-corrected SERS signals *vs* spiked DA concentration (color scale) (b) with Fe^III^ omitted, (c) *PostNP* PSALM assay, and (d) *PreNP* PSALM assay. Gray lines show negative control spectra using water as the analyte. (e) Scores of first-principal component from three repeats of spectra in panels (b)–(d) *vs* spiked DA concentrations. (f) Log–log plot of panel (e), with noise levels indicated as 2σ.

The fractal architecture of the aggregates efficiently confines light to hundreds of hotspots within each aggregate (through extended optical “chain modes”^33,34^). However, the precise assay protocol is important for ensuring that as many analyte molecules as possible end up in the spatially localized hotspots where they can be detected.^31^ To optimize the PSALM signals, we compare two protocols, *PreNP* and *PostNP* (Figure 1a). For any hope of translation into widespread use, it is essential to employ quality-assured commercial nanoparticles with long shelf life, and citrate-capped NPs are the most reliable such product available in bulk. Fe^III^ here plays a critical role in bridging NTs of interest with citrate, which is the charge-stabilizing surfactant used on the AuNPs (see the Methods section).

The difference between our two protocols is the order of addition of Fe^III^ in the formation of each PSALM. In *PreNP*, Fe^III^ is precoated onto AuNPs before aggregation and the sample solution containing NTs is added later. When 100 μM of Fe^III^ is added, the zeta potential on the AuNPs increases as the Fe^III^ interacts with the citrate (see Table S1). Higher concentrations of Fe^III^ result in unwanted Fe^III^-induced aggregation of AuNPs through screening-induced reduction of their Coulomb barriers, which dramatically decreases the ability of Fe^III^ to capture NTs into hotspots (Figure S3). In *PostNP*, AuNPs are first aggregated by NaCl, followed by the addition of the sample solution, which includes complexes of Fe^III^ and NTs (see below). Comparing the extinction spectra during aggregate formation for *Pre*/*PostNP* protocols (Figures S4 and S5; including corresponding SEMs) shows that precoating the AuNPs with Fe^III^ slightly slows aggregation, giving less red-shifted peaks, which can thus slightly reduce the resulting SERS. Here, 785 nm excitation is chosen as it enhances in both in-coupling and out-coupling, though 830 nm excitation would also be suitable. Initially, DA is used as our test molecule, selected from the set of targeted NTs. Comparing the SERS intensity with increasing [DA] shows that the effect of Fe^III^ is dramatic (Figure 1b–d). With Fe^III^ omitted, SERS signals increase at most 10-fold with the addition of DA. This suggests that although DA is likely to adsorb onto citrate via electrostatic attraction, the affinity between DA and citrate is weak. A much stronger binding between Fe^III^–citrate and Fe^III^–DA suggests that it provides an improved way to bind DA to the surface of AuNPs,^35^ thus giving SERS signals thousands of times larger with Fe^III^ included (Figure 1c,d). As we discuss later, additional factors may also contribute to this massive enhancement in the DA signal, including driving forces that guide DA into the gaps between AuNPs (through solvophobic interactions).

The optimal extraction of signal strengths requires data processing of these spectra, and in applications has to be unsupervised. Typically, the background of SERS spectra is complicated and less consistent. Rather than performing background fitting and subtraction to each spectrum, we utilize principal component analysis (PCA). These principal components represent linearly transformed eigenspectra that have different levels of correlation to the original SERS spectra, with corresponding scores (weights of eigenspectra). A complete set of eigenspectra and scores reconstructs the original SERS spectra.^36^ By using this transformation procedure, extracted scores can be defined, which are zero for samples without DA, and give values characteristic of the SERS strengths (Figure 1e). A suitable regression model for the data is a Langmuir–Hill fit, which represents receptor (Fe^III^-lined nanogaps) and ligand (NTs) systems (see Tables S2 and S3 for fitting coefficients). From the fit of the first-principal component (see Figures S7 and S8 for variance contributions and PCA loadings), it is possible to determine the LODs for the different Fe^III^ aggregation methods (as well as limits of quantitation). Here, LODs are defined at the intersect of the Langmuir–Hill fit and the 2σ confidence band of the noise level (asymptote for *c* → 0). This method is a rather conservative estimate of the LOD. Not surprisingly, the assay without Fe^III^ has the lowest score even at high DA concentrations and it saturates above 1 μM, with the best LOD of ∼100 nM. Since this is not enough for the clinical range demanded, it shows that despite being an efficient SERS substrate for a wide variety of other molecules, NTs are not able to access the hotspots and thus further advancement is required.

Incorporating Fe^III^ in the assay delivers this required enhancement, with *PreNP* PSALM giving the lowest LOD of ∼1 nM, while *PostNP* gives an LOD of ∼12 nM (Figure 1f). Usefully, *PreNP* has also a wider dynamic range of measurable DA concentrations than *PostNP*. This suggests that *PreNP* PSALM provides a more accessible pathway for DA to attach to the gap surfaces of aggregated AuNPs. Precoating Fe^III^ on AuNPs may support surface attachment followed by surface migration into hotspots. By contrast, as we now discuss, in the *PostNP* protocol, DA already exists in solution as a mono-, bis-, or tris-complex with Fe^III^. This may account for the lower dynamic range from *PostNP* PSALM as steric effects can then limit the fraction of Fe^III^-bound DA, which binds to the AuNPs and migrates into the hotspots.

### Characterization of Fe^III^–DA Complexes in Solution at Various pH Values

To better understand the key role played by the Fe^III^ complexation in enhancing this NT assay, a characterization is performed on the complexes formed between the Fe^III^ and DA in the preincubation stage of *PostNP*.

**Figure 2:**
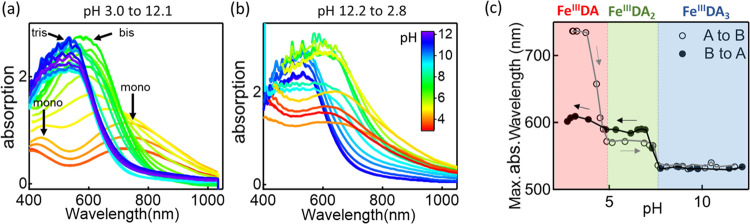
Evolution of the absorption spectra of the complexes of DA and Fe^III^ in (a) acidic to basic (A to B, red to blue) and (b) basic to acidic (B to A, blue to red) pH titration. (c) Extinction spectra peak wavelengths from panels (a, b) *vs* pH. Regions where Fe^III^DA, Fe^III^DA_2_, and Fe^III^DA_3_ dominate are shaded red, green, and blue.

According to the literature, for Fe^III^/DA complexes the monocomplex has absorption peaks at 406 and 759 nm, while the bis- and tris-complexes have single peaks at 575 and 492 nm, respectively.^37^ Extinction spectra of Fe^III^/DA complexes (DA without Fe^III^ is shown in Figure S6) are thus measured, while the solution is titrated from a low pH to a high pH (Figure 2a) and *vice versa* (Figure 2b). The fraction of these species in different solution pH values can thus be extracted (Figure 2c), resolving three regions from the step-like curve (shaded colors denote Fe^III^DA, Fe^III^DA_2_, and Fe^III^DA_3_ regions). Interestingly, pH titration from base to acid does not completely reverse to the original acid state. This is likely due to irreversible oligomerization between DAs bound via Fe^III^ at high pH. Several hours are needed for full DA polymerization at pH > 10,^37^ so during the 30 min measurement here DA disassociated from Fe^III^ partially oligomerizes and cannot return to the monomer even at low solution pH. We note that the Fe^III^–NT binding strength is 6 nN,^35^ so after formation and without changing pH, these complexes are stable. This binding between Fe^III^ and NTs is thus relevant to the incorporation of DA into plasmonic hotspots.

### Raman Scattering of Fe^III^–DA Complexes and Their SERS by PSALM

**Figure 3:**
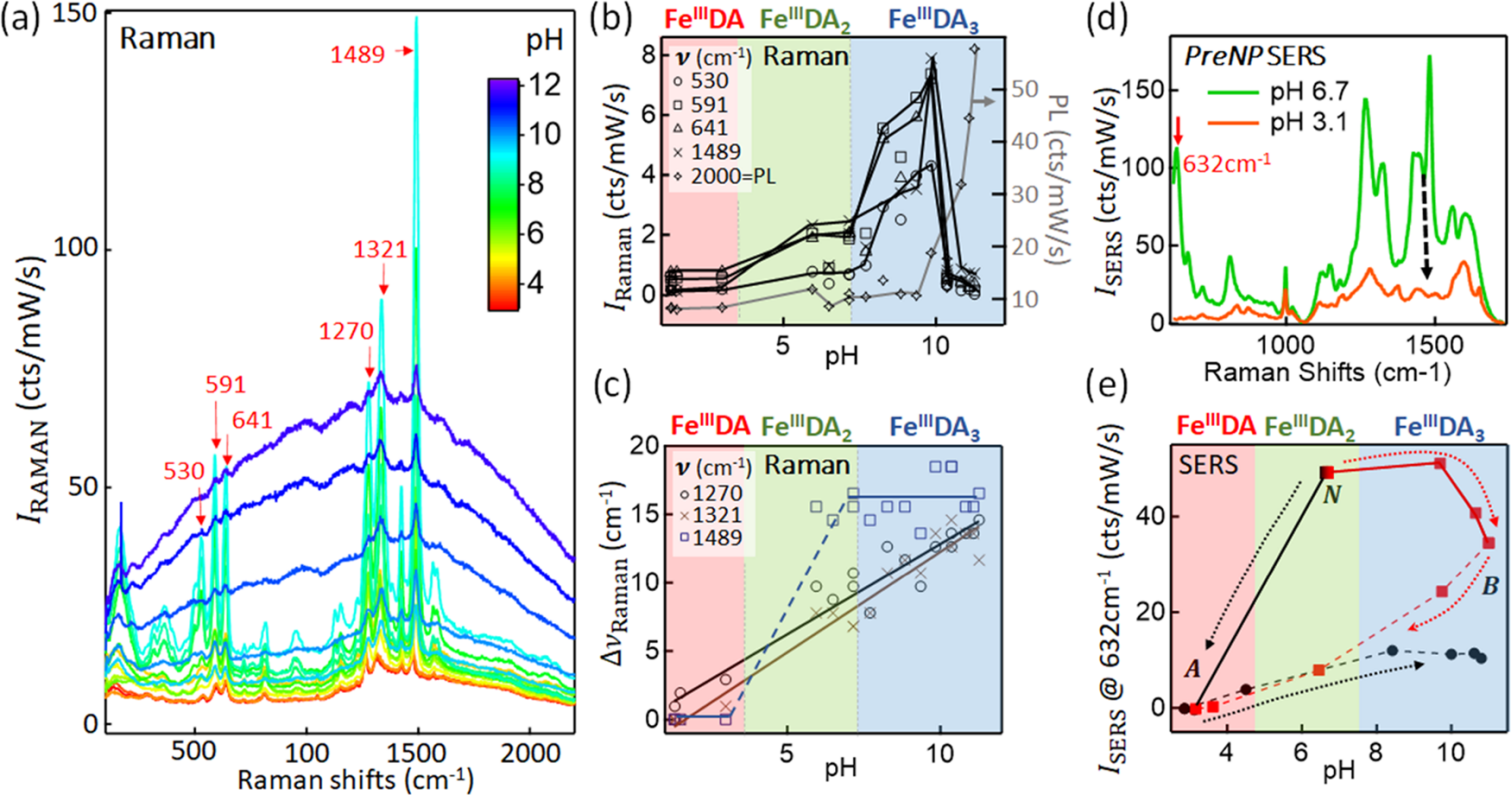
(a) Evolution of Raman signals from Fe^III^–DA complexes *vs* pH at 50 mM DA with 50 mM Fe^III^. (b) Raman intensity of 530, 591, 641, 1270, and 1489 cm^–1^ lines (arrows in panel a) and the photoluminescence background (measured at 2000 cm^–1^), as solution pH changed from acid to base. (c) Shift of Raman peaks initially at 1270, 1321, and 1489 cm^–1^*vs* pH. Red, green, and blue regions indicate Fe^III^DA, Fe^III^DA_2_, and Fe^III^DA_3_ dominating species. Lines are guides to the eye. (d) SERS of DA obtained from *PreNP* PSALM at pH 3.1 and 6.7. (e) SERS intensity of DA at 632 cm^–1^ as pH changed from neutral (*N*) to base (red squares, then to acid, dashed) and from *N* to acid (black circles, then to base, dashed).

Despite the clear involvement of Fe^III^ in bringing DA into the plasmonic gaps, it is so far unclear in which form it is bound. Raman spectroscopy at 785 nm is thus now used to check the gap contents. Initially, we calibrate the Raman spectra of the Fe^III^–DA complexes in solution without AuNPs (Figure 3a). The covalent coordination binding is through lone pair electrons between the central Fe^III^ and the deprotonated hydroxyls of DA^38^ (Figure 1a). The Raman intensities increase significantly with increasing solution pH up to 10, particularly for peaks at 530, 591, 641, 1270, 1321, and 1489 cm^–1^ (all absent without DA). This directly tracks the amount of DA bound to each Fe^III^, as seen in Figure 3b. At lower pH, a higher fraction of the hydroxyl groups is protonated preventing their coordination to Fe^III^, leading to a far weaker Raman intensity. This is surprising since the 785 nm laser is near-resonant with electronic transitions seen in absorption at low pH (Figure 2a,b), which should give resonant Raman and must be due to much higher Raman cross sections for Fe^III^DA_3_, from electronic delocalization across the complex. By contrast, the Raman intensity drops suddenly for pH > 10, which is likely due to polymerization between DAs (oligomerization).^37^ The fluorescence generated by these newly formed oligomers is seen as an increasing background under the Raman spectrum (Figure 2b, 2000 cm^–1^). Small spectral shifts (Δν <1%) of the Raman peaks (Figure 3c) show Δν ∝ pH for 1270 and 1321 cm^–1^ but reveal a jump at pH > 7 and < 7 for the 1489 cm^–1^ ring vibration mode (catechol ring).

Compared to the concentrated 1 M DA solution required to capture these Raman measurements (LOD ∼ 100 mM), aggregated AuNPs are vital to provide SERS enhancement when the analyte concentration is very low, <1 μM. SERS spectra are typically more complicated and shifted compared to Raman since signatures of surfactants on AuNPs (*e.g.* citrates) are also enhanced, while chemical shifts from bond hybridization with the metal also occur. Helpfully, SERS contributions from surfactants are insignificant here, and the SERS spectra of Fe^III^–DA complexes in AuNPs match their solution Raman spectra (Figure S9). As before, below pH = 4.5, DA protonation results in the partial dissociation of DA from the Fe^III^,^39^ eliminating the characteristic DA SERS peaks leaving only protonated surfactant molecule peaks^40^ (Figure 3d).

While pH-induced SERS peak shifts are absent, the SERS intensities change depending on the direction of the pH adjustment (Figure 3e). Initially, the colloidal AuNPs are buffered to pH 6.3 and are then adjusted to either acidic or basic conditions. For increasing pH, the SERS intensity drops irreversibly at pH > 10 (no recovery is observed for subsequent pH → 3). Minimal SERS increase is seen from pH 6.3 to >7.5, in contrast to the Raman measurements (Figure 3b). This suggests that in PSALM, the higher Raman cross-sectional Fe^III^DA_3_ complex is unable to form, perhaps because at least one of the available DA binding sites on Fe^III^ is occupied by the surfactant citrate at the AuNP’s surface, which is yet to be directly experimentally confirmed. Permanent oligomerization between DAs bound to Fe^III^ for pH > 10 seems to dramatically reduce their Raman cross section, perhaps by detaching them from SERS-enhancing Fe^III^. On the other hand, initial acidification to pH 3 protonates the DA, cleaving it from Fe^III^ causing the SERS to disappear. It also seems to disassociate the Fe^III^ from citrate on the AuNPs because even returning to pH 6 only restores <10% of the signal, suggesting that the active sites in the gap are now locked up. The optimal pH condition for the *PreNP* PSALM assay is identified to be around pH ∼ 7–8, where multiple DA complexation with Fe^III^ is favored.

### Proposed Mechanisms for Binding of Fe^III^ and NTs in PSALM

**Figure 4:**
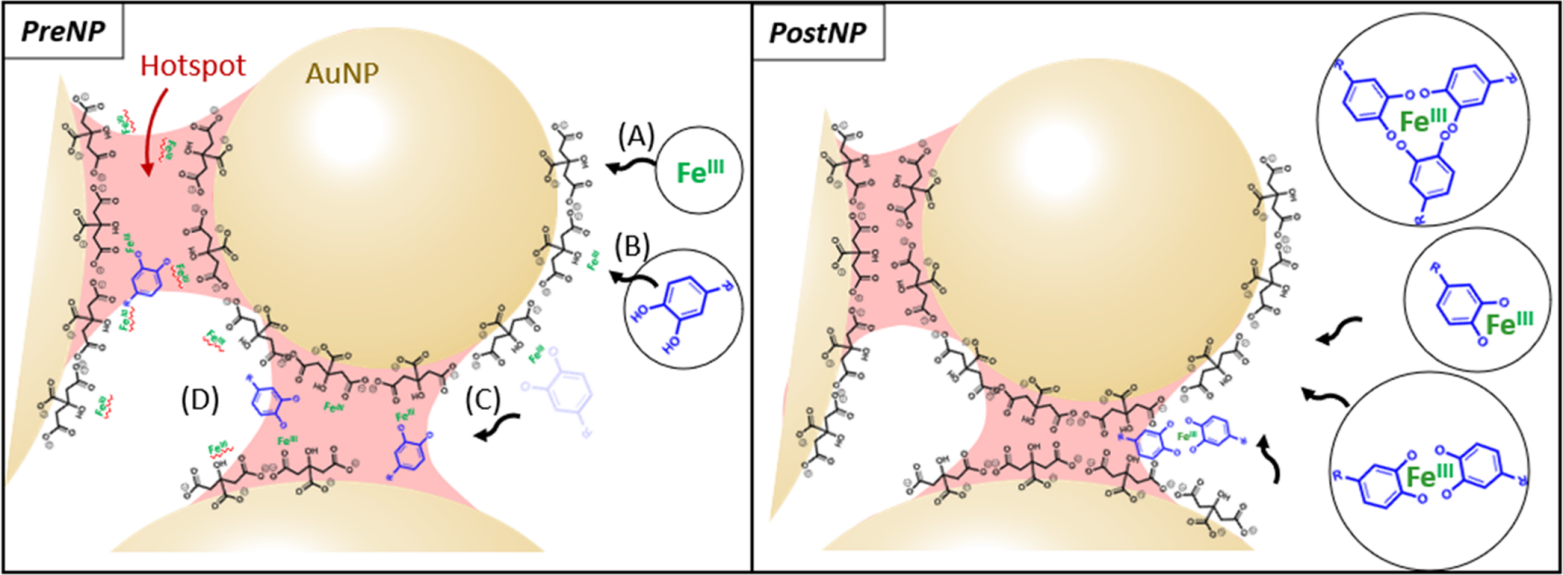
Proposed sequestration of NTs in optical hotspots between aggregated AuNP comparing *PreNP* and *PostNP* PSALM. In *PreNP*: (A) Fe^III^ chelates surfactant citrate on AuNPs, (B) NTs diffuse to these sites and chelate to Fe^III^, (C) Fe^III^NT complex migrates to hotspots. In *PostNP*: Fe^III^DA, Fe^III^DA_2_, and Fe^III^DA_3_ formed in solution diffuse to citrates and migrate to the hotspots. (D) Single NT can potentially chelate with two Fe^III^ only in the gap regions.

These characterizations of PSALM efficacy for *PreNP* and *PostNP* protocols, and the complexation of NTs and Fe^III^, allow for a detailed discussion of the mechanisms by which NTs reach the hotspots after they are added to AuNP aggregate solutions (Figure 4).

In the *PreNP* scenario, preincubation allows each Fe^III^ to bind to citrate on the AuNPs (the surfactant used in typical synthetic routes for AuNPs). This allows NT molecules to attach to the AuNP surface by binding to the citrate-complexed Fe^III^ on the Au. It then appears that these complexes migrate across the surface into the hotspots to give an LOD of ∼1 nM DA in water. This hypothesis is favored by recent work using a similar platform where AuNP aggregation is achieved through cucurbit[5]uril complexation (CB[5]).^41^ In that system, ethanol and methanol migrate to the more hydrophobic hotspots between AuNPs bridged by CB[5]. It is also possible that hydroxyls can link two Fe^III^ in the hotspot (Figure 4, process D), provided Fe^III^ covers AuNPs densely (here 2.3 × 10^6^ Fe^III^ per AuNP, so up to 200 Fe^III^ per nm^2^ of Au).

By contrast, in the *PostNP* scenario, complexes of Fe^III^ and NTs form in advance of addition to the aggregate solution. These presumably bind to the AuNP surface and hence into the hotspots by a similar mechanism as in *PreNP*. The high fraction of Fe^III^NT_2_ in *PostNP* PSALM, which is buffered to pH 6.3, contrasts with *PreNP* PSALM where individual NTs diffuse onto the aggregates. *PreNP* has a nearly 100-fold lower LOD but gives only 50% of the SERS strength of *PostNP*. These effects likely result from the complex binding, multiple chelation, and surface migration mechanisms involved.

### SERS and LOD for Different NTs in PSALM

**Figure 5:**
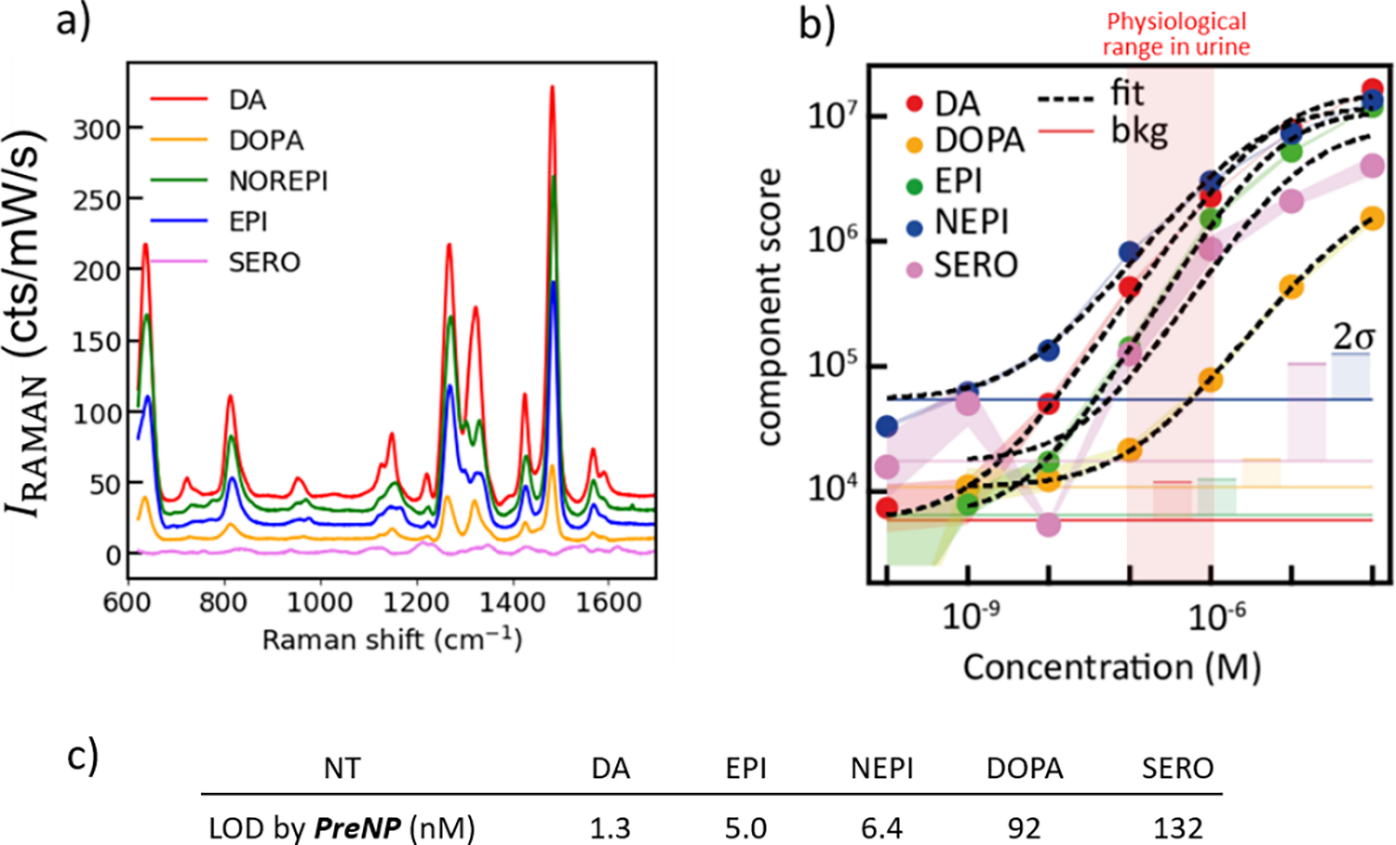
(a) Calibrated SERS intensity (cts/mW/s) of NTs, where NEPI, EPI, DOPA, and SERO are norepinephrine, epinephrine, l-DOPA, and serotonin, respectively (offset for better visualization). (b) Scores of first-principal components for NTs in PSALM using *PreNP* on linear and log plots. Lines are fits to the Hill–Langmuir equation. The shaded red region gives physiological concentrations of NTs in urine. (c) Measured limits of detection (LODs) of NTs using *PreNP*.

The sensing of not only DA but also l-3,4-dihydroxyphenylalanine (l-DOPA), epinephrine (EPI), norepinephrine (NEPI), and serotonin (SERO) was tested using PSALM (Figure 5a). Considering that the molecular structures of these NTs (Figure S1) resemble each other, except for different functional groups on the other end of the hydroxyl NT terminus, it is clear why their SERS spectra are similar (though distinguishable). Since there is only one hydroxyl bond in SERO, while two in the other molecules, it is most likely that the weaker SERS intensity of SERO is due to weaker binding. This again emphasizes how bidentate Fe^III^ chelation is vital to this assay. This means that PSALM can potentially be extended to NT metabolites, which still contain two *ortho*-dihydroxyls such as 3,4-dihydroxyphenylacetic acid. Other important metabolites, *e.g.* 3-methoxytyramine and homovanillic acid, will most likely not undergo Fe^III^ enhancement, similar to SERO (as they lack the *ortho*-dihydroxyl groups that facilitate the strong binding to Fe^III^).^42,43^

The low LOD for *PreNP* PSALM suggests that the unassisted solution diffusion of soluble NTs to the hotspots followed by chelation to localized Fe^III^ in the hotspot provides an efficient assay. To compare these assays, PCA scores for DA, DOPA, NEPI, and EPI are obtained from *PreNP* PSALM *vs* NT concentration (Figure 5b and Table S3). Despite their chemical similarity, both signal strengths and LODs (Figure 5c) are significantly different. For NTs with two *ortho*-hydroxyls, LODs of DA, EPI, and NEPI are much lower than DOPA; while DA has the highest signal strength, DOPA is the lowest of this set. This implies that whether or not the functional groups in an NT have net charge influences their ability to diffuse onto the surface of AuNPs in *PreNP* PSALM and subsequent migration to the hotspots. The reason that l-DOPA has the worst LOD and signal strength among NTs with two hydroxyls probably results from the additional negative charge of the carboxylate (which the other NTs do not have), which is repelled from the negative surface charge of the citrate-coated AuNPs.

From the results shown here, we suggest that the Fe^III^ ions are efficiently pulled into the hotspots through attraction to locally concentrated negative citrate ligands in this confined region. In turn, these Fe^III^ are efficient at strongly binding the NTs into the same location, where they are optimally positioned for high signal SERS sensing. All small hydroxyl species in urine will be captured in this way, and thus we now study competition for the hotspot sites.

### Sensing NTs in Urine by PSALM Using Pretreatment through Affinity Separation

The aim of PSALM is to detect NTs quantitatively in biofluids, so urine is adopted for testing its efficacy. Since urine contains numerous ions, small molecules, peptides, and cells in water, it is not surprising that the interference of Fe^III^ binding with unwanted components other than NTs in urine (particularly creatinine) significantly reduces the ability of PSALM to detect NTs (Figure S3). Determining every possible species in urine that interferes with the Fe^III^ binding assay and then establishing filtration methods for each is unfeasible and costly. Therefore, rather than filtering out the many interferants that can be present in urine, here we sift out the targeted NTs from the urine directly.

**Figure 6:**
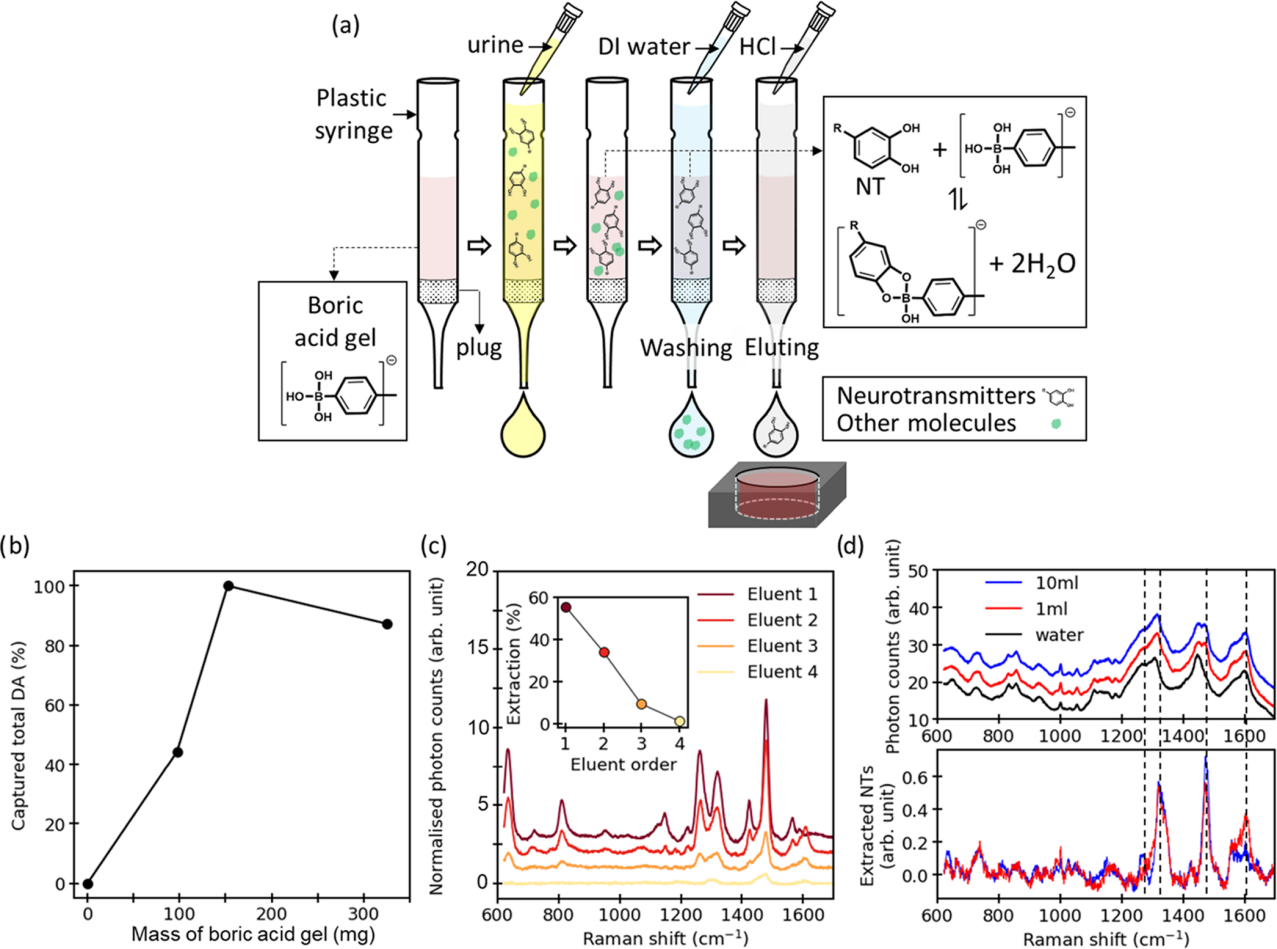
(a) Affinity separation procedure for extracting NTs via their retention on boric acid gel. The retained NTs are sequentially released for PSALM using successive HCl eluents. (b) The percentage of total captured+released DA *vs* mass of boric acid gel used in the column. (c) Normalized PSALM SERS (*PreNP*) from a sample of 1 mL containing 10 μM DA after affinity separation into eluent fractions. The inset graph shows the percentage of DA extracted in each eluent fraction. (d) Normalized PSALM SERS (*PreNP*) of 2 mL first eluent from affinity-separated 1 and 10 mL samples of fresh urine through 174 mg of boric acid gel. The lower graph shows extracted PSALM NT spectra (after subtracting water response), after baseline correction. Note that eluent is pH-buffered with NaOH to pH 6.5–7.5 for PSALM.

Affinity chromatography for NTs using boric acid gel has been previously explored.^44^ We find that the NTs from urine are retained on a boric acid gel (Figure 6a), and other unwanted interferant molecules are washed away with water. The gel-retained NTs can be released efficiently using an acidic mobile phase (HCl), but the collected fractions have to be adjusted back to pH 6.5–7.5 to optimize PSALM (Figure 3e). We determined (Figure 6b) that 150 mg is the minimum mass of boric acid gel that can retain 97 ± 3% of 10 μM DA (the upper limit of physiological concentrations of NTs in urine) in 1 mL of water (which is the typical volume of samples in a urine bank). All of the DA can be extracted in four eluents of 1 mL of 0.025 M HCl, with 90 ± 3% of DA in the first two eluents (Figure 6c). Using a single 2 mL release elution thus optimally collects most NTs and avoids decreasing SERS signals through excessive dilution. The characteristic SERS peaks of NTs observed in fresh urine (Figure 6d) include contributions from catecholamines (NTs with a pair of hydroxyl groups) with little else observed. The extracted NT signal shows no significant difference between 1 and 10 mL urine samples when using 174 mg boric acid gel, showing that the collection can be saturated. The next stage is to establish a more comprehensive database that allows a full deconvolution and quantification of NTs in urine (using various techniques such as artificial neural networks^45^ or machine learning^45^). However, in this single 2 mL eluent, we can already directly quantify the total NT load, estimated for this sample to be 125 nM (Figure 6d). We confirm that running the assay twice on the same urine sample gives results within 10% and that washing the boric acid gel allows it to be repeatedly reused (life cycle analysis in progress). We also confirmed that the source of Fe^III^ is irrelevant to the PSALM assay (iron nitrate or iron chloride; Figure S10). We evidence here a full assay from normal urine samples that quantifies the NT load and paves the way to low-cost real-time monitoring of health-critical biomolecules. The improved LOD over conventional SERS makes this technique comparable to the typical best LODs of competing methods (see Table S4).

## Conclusions

In conclusion, we have developed a liquid platform for sensing neurotransmitters through a PSALM assay. This quick and simple “mix-and-measure” method enables the detection of multiplexed NTs at and below the physiological range of concentrations in water and in urine following suitable pretreatment by affinity separation. Such an assay forms the only way to collect long time-series measurements in normal settings such as at home and clinic and opens a completely new measurement space, which has not been accessed previously—indeed, nothing is currently known about NT fluctuations over days, months, or years or how this is influenced by diet and drug regimens.

This suggests that long-term monitoring of mental stress and mental illness through sensing NTs in biofluids may become possible for the first time through PSALM. We showed that two different protocols, *PreNPvsPostNP*, are effective but give different LODs and signal strengths. In general, LODs from *PreNP* PSALM are significantly lower than those of *PostNP* PSALM. Our experiments confirm the importance of Fe^III^–NT complexes, which depend on pH, and suggest that Fe^III^DA_2,3_ have larger Raman cross sections while monomeric Fe^III^DA more easily migrates into the hotspot gaps. By varying pH, protocols, and reagent concentrations, we identify the most crucial factors in NT PSALM sensing and show optimal conditions.

## Methods

### Chemicals

Sodium chloride (NaCl), iron(III) nitrate nonahydrate (Fe(NO_3_)_3_·9H_2_O), dopamine hydrochloride (C_8_H_11_NO_2_·HCl), epinephrine hydrochloride (C_9_H_13_NO_3_·HCl), norepinephrine hydrochloride (C_8_H_11_NO_3_·HCl), dihydroxyphenylalanine (l-DOPA, C_9_H_11_NO_4_), serotonin hydrochloride (C_10_H_12_N_2_O·HCl), hydrochloric acid (HCl), sodium hydroxide (NaOH), and boric acid gel (184454–5mL) were purchased from Sigma-Aldrich and used without further treatments. Batches of 60 nm AuNPs (100 mL, citrate stabilized) were purchased from BBI Solutions.

### UV–Vis Extinction Spectroscopy

A disposable plastic cuvette with a light path of 10 mm (volume 1.5–3 mL, Sigma-Aldrich) filled with a liquid sample was fixed in a cuvette holder, illuminated by optical fiber-guided (200 μm, Ocean Optics) collimated ultraviolet–visible (UV–vis) light (360–2600 nm SLS201L, Thorlabs) traveling through the cuvette below the liquid surface of the sample. The transmitted light was collected by an identical optical fiber and was guided to the entrance of a spectrometer (QE65000, Ocean Insight). The transmission spectrum (*T*_s_), after subtracting the dark spectrum (*D*_s_) and taking DI water as the reference (*R*_s_), was converted to the extinction spectrum (*E*_s_) by
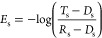


### Protocols of *PreNP* and *PostNP*

#### PreNP

Two microliters of 100 mM Fe(NO_3_)_3_·9H_2_O was first preincubated with 1998 μL of 0.288 mM of 60 nm AuNPs for 10 min to form complexes of Fe^III^ and citrate on the surface of AuNPs in an Eppendorf tube. Two hundred and fifty microliters of these Fe^III^-coated AuNPs was aggregated with 50 μL of saturated NaCl for 5 min in a black polypropylene 96-well microplate (Greiner Bio-One Ltd.). Subsequently, one or mixed types of NTs in water or pure urine was pipetted into the well and mixed thoroughly and then left for 5 min. Finally, 23.7 mW of 785 nm laser was loosely focused at an optimized depth below the liquid surface in the well and the acquisition of SERS was integrated for 10 s.

#### PostNP

Similarly, 10 μL of 45 mM Fe(NO_3_)_3_·9H_2_O was preincubated with 10 μL of NTs and 980 μL of DI water for 10 min to form complexes of Fe^III^ and NTs in an Eppendorf tube. Two hundred and fifty microliters of 0.288 mM of 60 nm AuNPs was aggregated with 50 μL of saturated NaCl for 5 min in a well microplate. Afterward, the complex of Fe^III^ and NTs was pipetted into the well and mixed thoroughly, and the sample solution remained undisturbed for 5 min. Consequently, 23.7 mW of 785 nm laser was used to excite the solution at an optimized focusing depth and SERS was collected for 10 s.

### Raman Microscopy

Measurements of Raman scattering used an inVia confocal Raman microscope. The 785 nm laser was first filtered by a clean-up filter, followed by reflection at a dichroic beam splitter and then focusing through a 5x objective (NA = 0.2, Nikon) to excite from the above liquid samples loaded in a 96-well microplate, which was placed on a motorized 3-axis microscope stage. Simultaneously, the scattering Raman signal from the excitation spot was collected through the same light path traveling back through the objective, passing through the dichroic beam splitter, dispersed by a grating, and focused on a TE-cooled back-thinned charge-coupled device (CCD) sensor collecting spectra on a computer for postanalysis. The optimized depth below the surface of the liquid sample for laser excitation was determined by finding the maximum strength of the Raman signal by automated *z*-axis scanning.

### Affinity Separation

A 1 mL disposable plastic syringe was filled with boric acid gel of an optimized weight depending on the targeted concentration of NT extractions (Figure 6b), in which a 10 mm height of glass wool was preloaded as a plug, close to the pipette tip. Ten milliliters of 0.025 M HCL was passed through first to guarantee the minimal unspecific adsorption in the column, and then pH 7.4 0.1 M phosphate buffer was used to activate the boric acid gel. One milliliter of DA dissolved in water or 1 mL of urine was added to the syringe, where the boric acid gel captures NTs specifically and allows most of the rest of the molecules to pass through. The remaining unwanted molecules were then washed away by using 10 mL of DI water. Eluent fractions released by 25 mM HCl were collected and then pH-adjusted to 6.5–7 for the next-stage SERS measurements.

### Principal Component Analysis

PCA is applied to analyze the concentration series data for SERS spectra.^41^ By using PCA, we accurately identify and quantify the individual analyte components and correlate SERS intensities with analyte concentrations to extract information. PCA is a standard mathematical method that linearly transforms data sets onto orthogonal eigenvectors, which are arranged in order of correlation between data points. The first eigenvector, also representing the direction of the first-principal component, is of the most significant variation in data sets. Scores are projections of original data sets onto transformed axes, and they characterize how principal components correlate to the original data set. This is particularly useful when we scan the concentration *m* of one analyte. In the case of this series of spectra, each component consists of an “eigenspectrum” *E*_*i*_(ν) (a set of spectral components, which change together) and a weight *c*_*i*_ (often known as a PCA “score”), which gives the contribution of its associated eigenspectrum to the measured spectrum. The contribution *y*_*i*_ of a single component to the full spectrum at concentration *m* is then: *y*_*i*_(*m*) = *c*_*i*_(*m*)*E*_*i*_. Combining all of the contributions yields the counts at each wavelength, reconstructing the measured spectrum
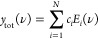
where *y*_tot_ is the SERS emission at wavenumber ν. A practicality regarding PCA is that the physical meaning of the eigenspectra can only be attributed to additional knowledge of the experimental system.

PCA is used to exploit the change in spectra upon analyte addition to isolate the SERS contributions of the individual components from a concentration series. The eigenspectrum of the first component obtained using PCA (principal component 0, or *E*_1_) closely resembles the spectra of sample without analytes. The eigenspectrum of the second major component (principal component 1, or *E*_2_) is from the analyte since a steady increase in its corresponding scores is observed with the addition of analyte.

## Supporting Information Available

Molecular structures of NTs and their SERS spectra (Figure S1); SERS concentration series of DA using CB (Figure S2); molecular structures and SERS of urea, creatinine, uric acid, and mixtures with DA (Figure S3); extinction spectra of BBI 60 nm AuNPs precoated with a range of Fe^III^ concentrations (Figure S4); extinction spectra of BBI 60 nm AuNPs precoated with Fe(NO_3_)_3_ and FeCl_3_ (Figure S5); extinction spectra of pristine DA at different pH values (Figure S6); Zetasizer measurements (Table S1); Langmuir–Hill fit parameters, LODs, and LOQs (Tables S2 and S3); variance % for each PCA analysis (Table S4); PCA variance contributions for PreNP DA (Figure S7); PCA loading plots for PreNP, PostNP, and no Fe^III^ (Figure S8); Raman spectra of DA–Fe^III^ complexes at different pH values (Figure S9); SERS spectra of NTs with Fe(NO_3_)_3_ and FeCl_3_ (Figure S10); and list of acronyms (PDF)
