# Supplementary material for: Plasmonic Sensing Assay for Long-Term Monitoring (PSALM) of Neurotransmitters in Urine
Source: ACS Nanosci Au. 2022 Dec 24;3(2):161–71. doi: 10.1021/acsnanoscienceau.2c00048 (PMC10119978; doi:10.1021/acsnanoscienceau.2c00048)
Supplement: Supplementary file 1 — ng2c00048_si_001.pdf [file ng2c00048_si_001.pdf]

Plasmonic Sensing Assay for Long-term Monitoring (PSALM) of Neurotransmitters in Urine

Wei-Hsin Chen[1], Wenting Wang[1,2], Qianqi Lin[1], David-Benjamin Grys[1], Marika Niihori[1], Junyang Huang[1], Shu Hu[1], Bart de Nijs[1], Oren A Scherman[2], Jeremy J Baumberg[1,*]

[1] NanoPhotonics Centre, Cavendish Laboratory, University of Cambridge, J J Thomson Avenue, Cambridge, UK CB3 0HE

[2] Melville Laboratory for Polymer Synthesis, Department of Chemistry, University of Cambridge, Lensfield Road, Cambridge, UK CB2 1EW

Supplementary Information:

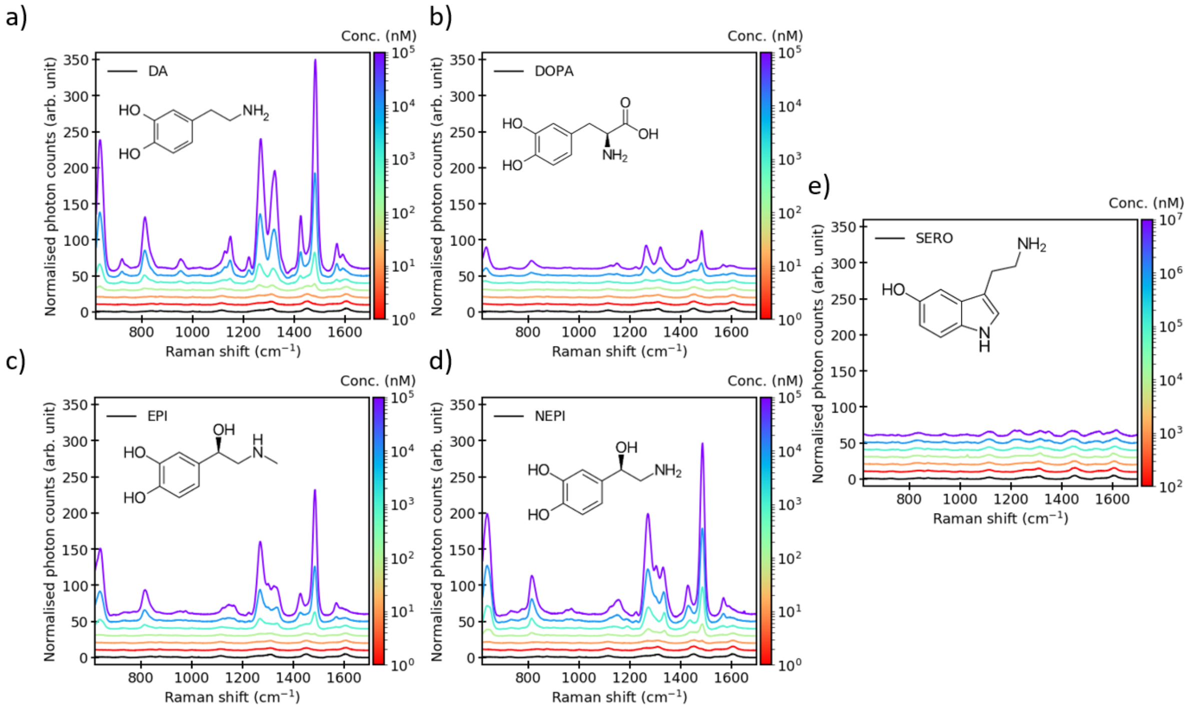

Figure S1 | Molecular structures and baseline-corrected SERS signals with Fe^{III} involved from 1 nM to 100 μM of (a) dopamine (DA), (b) DOPA, (c) epinephrine (EPI), (d) norepinephrine (NEPI) and (e) serotonin (SERO).

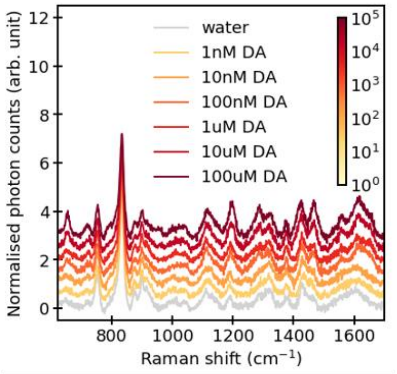

Figure S2 | Baseline-corrected concentration series of DA SERS signals from 1 nM to 100 µM with an optimised CB concentration of 200 µM.

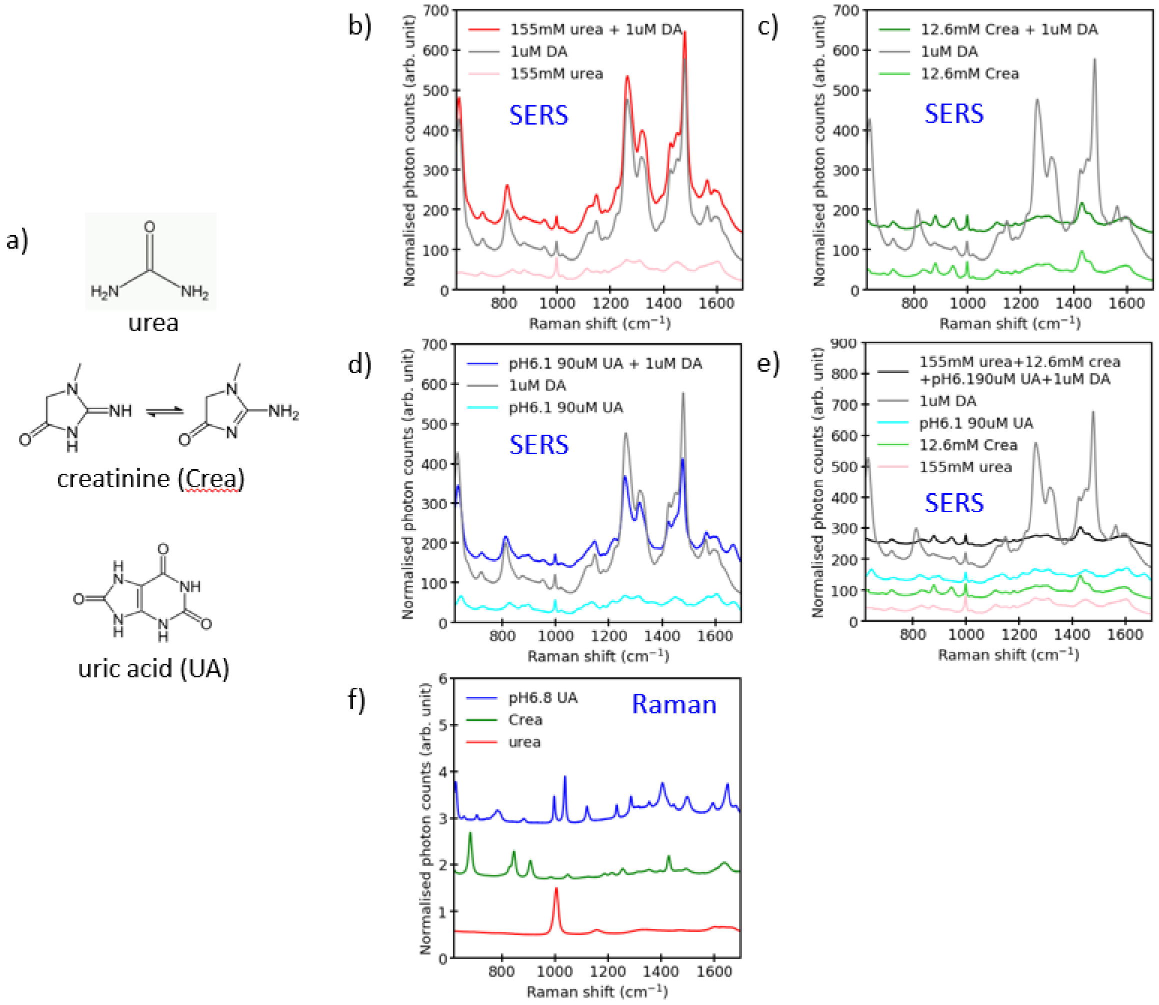

Figure S3 | (a) Molecular structures of urea, creatinine (Crea) and uric acid (UA). Baseline-corrected SERS signals with Fe^{III} involved of (b) urea, DA and their mixture, (c) Crea, DA and their mixture, (d) UA, DA and their mixture, (e) DA, urea, Crea, UA and their mixture. (f) Baseline-corrected Raman spectra (with AuNPs omitted) of urea, Crea and UA.

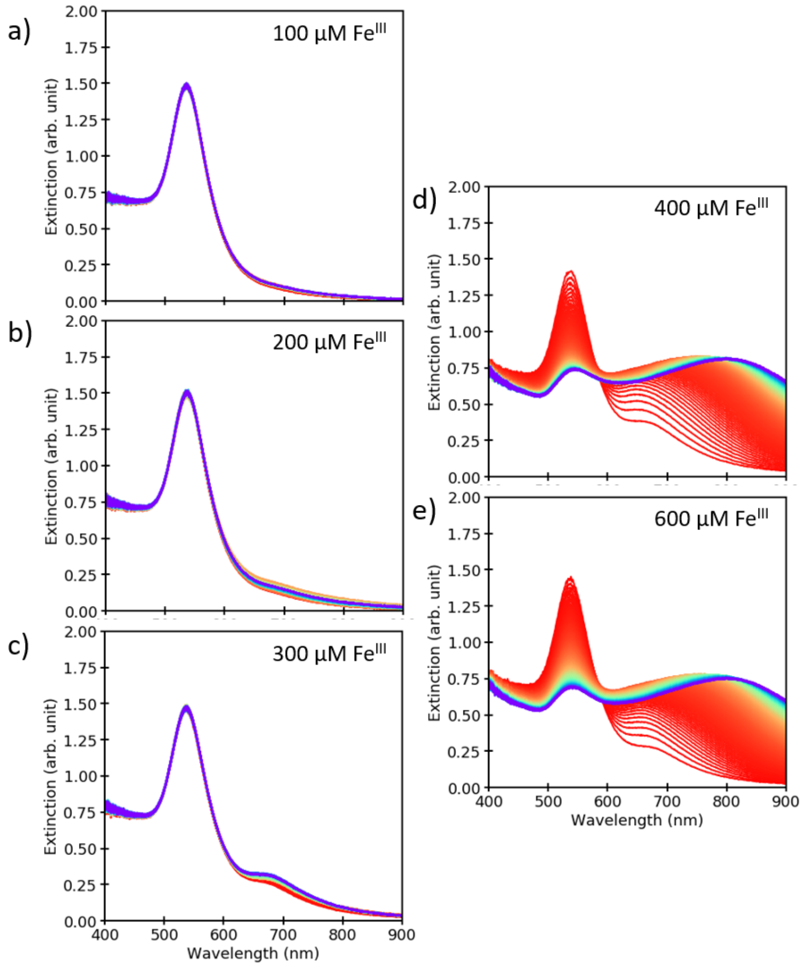

Figure S4 | UV-visible extinction spectra of BBI 60 nm AuNPs pre-coated with (a) 100 µM, (b) 200 µM, (c) 300 µM, (d) 400 µM and (e) 600 µM $Fe^{III}(NO_3)_3$. This shows that pre-coating with concentrations of Fe^{III} > 100 µM onto the AuNPs induces unwanted AuNP aggregation.

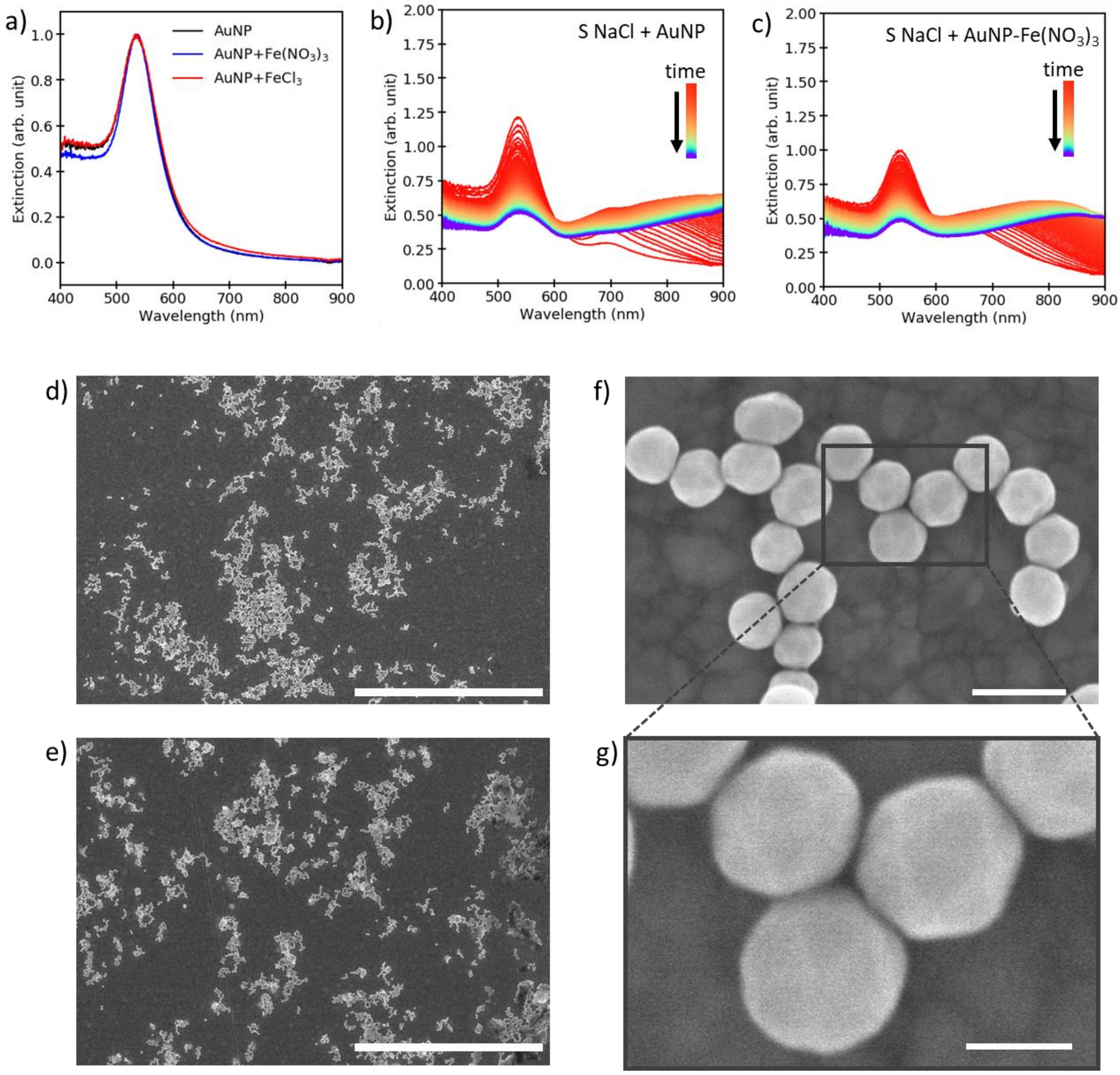

Figure S5 | UV-visible extinction spectra of (a) BBI 60 nm AuNPs, pre-coated with 100 µM $Fe(NO_3)_3$ and 100 µM $FeCl_3$. Saturated NaCl (S NaCl) induces (b) AuNP aggregation and (c) aggregation of AuNPs pre-coated with 100 µM $Fe(NO_3)_3$. SEM images of 60 nm AuNPs (d) pre-coated with 100 µM $Fe(NO_3)$ and aggregated with NaCl and (e) aggregated with NaCl without the presence of any Fe^{III}. (f) and (g) highlights that the gaps are retained by the NaCl aggregation and nanogaps do not collapse when AuNPs are pre-coated with Fe^{III}. Scale bars are 5 µm, 5 µm, 100 nm and 40 nm respectively in (d-g).

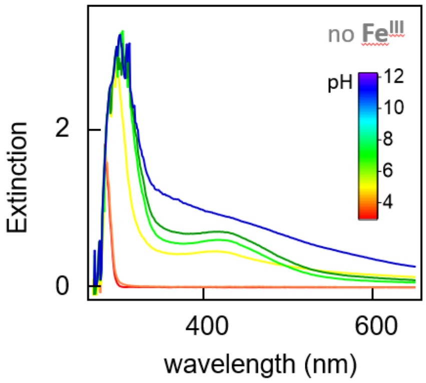

Figure S6 | Absorption spectra (with Fe^{III} omitted) of pristine DA at different pH values.

	AuNP (nm)	Zeta potential (mV)
**Without Fe^{III}**	66.11 ± 0.84	-18.13 ± 2.12
**With Fe^{III}**	67.75 ± 0.10	-9.90 ± 0.54

Table S1 | Zeta potential measurements of 60 nm AuNP without and with Fe^{III}, displaying both the size of the AuNPs and their zeta potential.

Langmuir-Hill model

The second components of the PCA in Figure 1e,f and Figure 5b are fitted according to the standard Langmuir-Hill equation

$$f(c) = log\left(b + A \frac{1}{1 + \left(\frac{K_a}{c}\right)^n} \right)$$

The key parameters of the fits are tabulated in Tables S1 and S2.

	**DA without Fe^{III}**	***'PostNP'* for DA**	***'PreNP'* for DA**
b [cts/mW/s]	3.96e+05 ± 3.02e+04	1.18e+05 ± 1.74e+04	6777 ± 1.01e+03
A	5.86e+09 ± 1.36e+11	2.95e+07 ± 7.77e+06	1.51e+07 ± 3.89e+06
K_a [M]	1.27e+08 ± 2.49e+09	3.51e+06 ± 2.45e+06	5.69e+06 ± 3.32e+06
n	1.18 ± 0.13	0.94 ± 0.14	0.94 ± 0.08
LOD [nM]	35	12	1.3
LOQ [nM]	123	59	6.6

Table S2 | Langmuir-Hill fit coefficients for Figure 1e,f, limit of detection (LOD: 2σ) and limit of quantification (LOQ; 9σ). Error is reported as one standard deviation.

	**DOPA**	**EPI**	**NEPI**	**SERO**
b [cts/mW/s]	1.809e+3± 2.35e+3	6519.4 ± 1.92e+03	47112 ± 1.06e+04	16490 ± 1.17e+04
A	7.8518e+06± 9.65e+07	1.10e+07 ± 2.57e+06	1.26e+07 ± 5.68e+06	8.49e+06 ± 5.44e+06
K_a [M]	1.5576e+09± 3.91e+10	6.91e+06 ± 3.51e+06	4.00e+06 ± 5.26e+06	1.88+07 ± 3.74e+07
n	0.58251± 0.31	1.04 ± 0.09	0.80 ± 0.17	0.93 ± 0.04
LOD [nM]	92	5.0	6.4	132
LOQ [nM]	122	21	42	698

Table S3 | Langmuir-Hill fit coefficients for Figure 5b limit of detection (LOD: 2σ) and limit of quantification (LOQ; 9σ). Error is reported as one standard deviation.

Analyte/preparation	Variance (%)
preNP DA	1.73
postNP DA	1.73
NoFE DA	1.70
DOPA	2.20
SERO	0.27
NOREPI	2.25
EPI	3.12

Table S4 | Variance in % of principle components representing an analyte

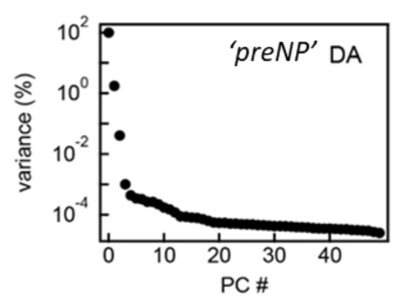

Figure S7 | PCA variance contributions after principle component decomposition of the *'preNP'* DA spectrum.

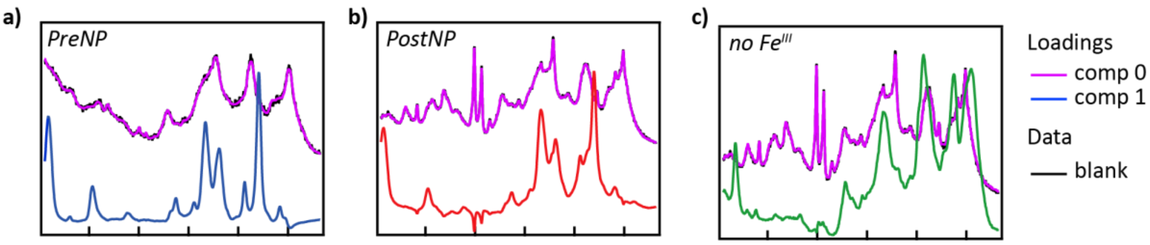

Figure S8 | PCA loadings of the first two components (comp0: black background) and compared to blank measurements without DA as background spectra (purple). a) *PreNP* protocol with comp 1 (blue) the DA response, b) *PostNP* with comp 1 (red) representing the DA loading, c) No Fe^{III} comp 1 (DA) response plotted in green.

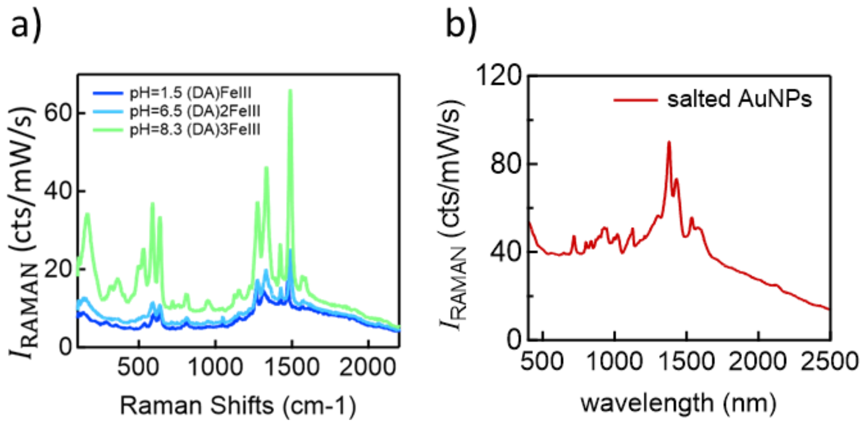

Figure S9 | (a) Raman spectra (with AuNPs omitted) of complexes formed by DA and Fe^{III}, including (DA)Fe^{III} at pH 1.5, $(DA)_2Fe^{III}$ at pH 6.5 and $(DA)_3Fe^{III}$ at pH 8.3. (b) Blank spectrum of AuNP, salt-aggregated at pH=7±0.5.

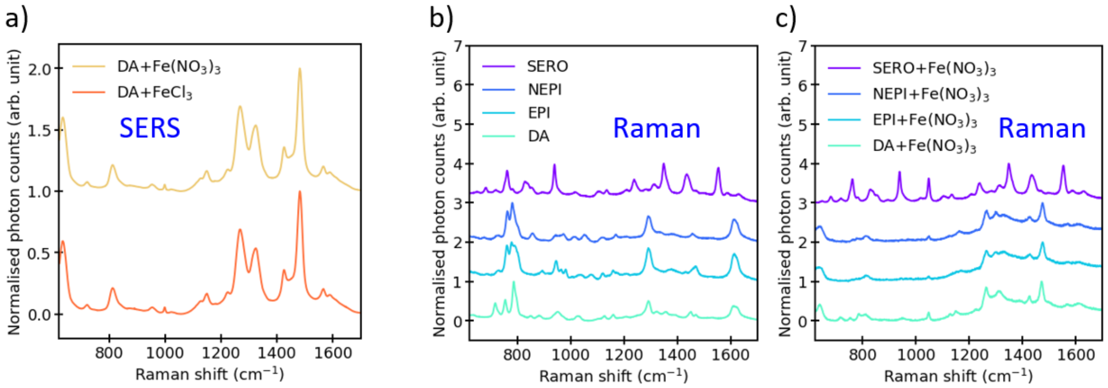

Figure S10 | (a) Baseline-corrected DA SERS signals with 100 µM Fe(NO$_3$)$_3$ and 100 µM FeCl$_3$. Baseline-corrected Raman spectra (with AuNPs omitted) of (b) 1 M SERO, NEPI, EPI and DA only in water and (c) 1 M SERO, NEPI, EPI and DA pre-mixed with 100 mM Fe(NO$_3$)$_3$.

DA sensing technique	LOD range (M)	
CE-EC	$9\cdot10^{-9}$	$1\cdot10^{-4}$
LC-EC	$5\cdot10^{-10}$	$1\cdot10^{-9}$
LC-MS	$2.5\cdot10^{-10}$	$1\cdot10^{-8}$
Microelectrode	$8\cdot10^{-9}$	$1\cdot10^{-5}$
Surface modified Microelectrode	$5\cdot10^{-9}$	$5\cdot10^{-5}$
SERS	$2\cdot10^{-8}$	$1\cdot10^{-6}$
PSALM	$1.3\cdot10^{-9}$	

Table S4: Comparison between LOD values reported in literature[1–3] for commonly used and emerging techniques for NT sensing.

List of acronyms

AuNP	Gold NanoParticles
DA	Dopamine
EPI	Epinephrine
L-DOPA	l-3,4-dihydroxyphenylalanine
LOD	Limit of detection
NEPI	Norepinephrine
NP	Nanoparticle
NT	Neurotransmitter
NTA	Nitrilotriacetic acid
PCA	Principal component analysis
PostNP	Protocol: AuNPs are aggregated by NaCl, followed by a sample solution of Fe^{III} and NTs
PreNP	Protocol: Fe^{III} is pre-coated onto AuNPs before aggregation
PSALM	Plasmonic Sensing Assay for Long-term Monitoring
SERO	Serotonin
SERS	Surface enhanced Raman spectroscopy
CB[5]	Cucurbit[*n*]urils

Supporting Information References

1. Vander Ende, E. *et al.* Physicochemical Trapping of Neurotransmitters in Polymer-Mediated Gold Nanoparticle Aggregates for Surface-Enhanced Raman Spectroscopy. *Anal. Chem.* **91**, 9554–9562 (2019).

2. Feng, L. *et al.* Self-Referenced Surface-Enhanced Raman Scattering Nanosubstrate for the Quantitative Detection of Neurotransmitters. *ACS Appl. Bio Mater.* **5**, 2403–2410 (2022).
3. Perry, M., Li, Q. & Kennedy, R. T. Review of recent advances in analytical techniques for the determination of neurotransmitters. *Analytica Chimica Acta* **653**, 1–22 (2009).
